# Visceral leishmaniasis complicated by hemophagocytic lymphohistiocytosis: A case report from a nonendemic area

**DOI:** 10.1002/ccr3.7309

**Published:** 2023-05-04

**Authors:** Anwar I. Joudeh, Hussein A. Elsiddig Awadelkarim, Mohammadshah Isam Gul, Mahmoud Salm Elayana, Dina Sameh Soliman, Aliaa Amer, Musaed Alsamawi

**Affiliations:** ^1^ Department of Internal Medicine, Al‐Khor Hospital Hamad Medical Corporation Doha Qatar; ^2^ Department of Internal Medicine, Hamad General Hospital Hamad Medical Corporation Doha Qatar; ^3^ Department of Laboratory Medicine and Pathology Hamad Medical Corporation Doha Qatar; ^4^ Department of Internal Medicine, Infectious Diseases Division, Al‐Khor Hospital Hamad Medical Corporation Doha Qatar

**Keywords:** acute medicine, allergy and immunology, infectious diseases

## Abstract

**Key Clinical Message:**

Visceral leishmaniasis and hemophagocytic lymphohistiocytosis share many features in common and may coincide in the same patient. Timely diagnosis and management of visceral leishmaniasis could save patients from unnecessary toxic treatment.

**Abstract:**

Visceral leishmaniasis and hemophagocytic lymphohistiocytosis share many clinical features in common and may coexist in the same patient. Visceral leishmaniasis should be promptly ruled out in patients coming from endemic areas before starting immunosuppressive therapy for hemophagocytic lymphohistiocytosis. The mainstay treatment, in this case, is anti‐leishmania medications preferably liposomal amphotericin‐B.

## 1 INTRODUCTION

Leishmaniasis is one of the most important neglected tropical diseases that is associated with significant morbidity and mortality.^1^, ^2^ The visceral form of leishmaniasis is mainly caused by two species namely *Leishmania donovani* and *Leishmania infantum* which are endemic in certain areas of the world including South Asia, East Africa, the Mediterranean region, and Brazil.^3^ In visceral leishmaniasis (VL), the protozoa accumulate and rapidly replicate within the reticuloendothelial system causing variable degrees of bone marrow suppression, hypersplenism, and hepatic dysfunction.

Hemophagocytic lymphohistiocytosis (HLH) is a life‐threatening disease resulting from dysregulated immune system hyperactivation.^4^ Although some patients with HLH are genetically predisposed to this condition, many cases are sporadic. Nevertheless, in either case, infection or altered immune hemostasis might trigger the episode of HLH.^4^ The mainstay of treatment for this disease is treating the underlying etiology if identified as well as immunochemotherapeutic agents and bone marrow transplant.^5^

VL and HLH do not only share many clinical manifestations in common, they frequently overlap in the same patient which might cause diagnostic dilemmas and treatment challenges.^6^ Herein, we describe the clinical course and management for a patient with VL complicated by HLH in a nonendemic area.

## 2 CASE PRESENTATION

**FIGURE 1:**
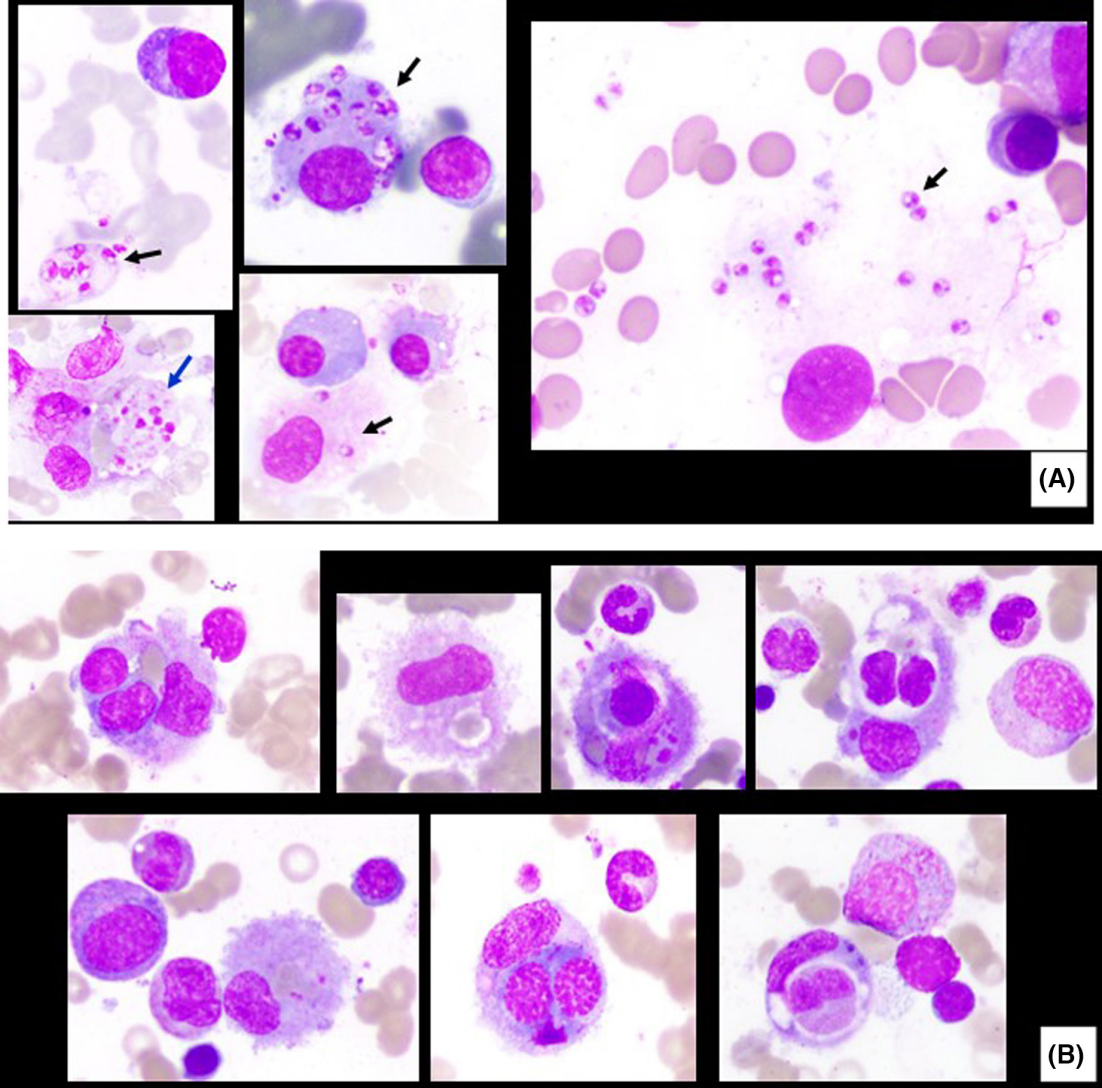
(A): Bone marrow aspirate (Wrights stain, 100×): Rare plasma cells and macrophages show extracellular and intracellular Leishman Donovan (LD) bodies (black arrow). Some tiny intracellular and extracellular particles also probably represent phagocytosed LD bodies (blue arrow). (B): Bone marrow histiocytes/macrophages showing active hemophagocytosis mostly erythrophagocytosis.

A 20‐year‐old Nepalese male with no previous significant medical history presented to the emergency department of a general hospital in Qatar with fever and anorexia for 1 week associated with episodic headache and vomiting. He denied any history of photophobia, skin rash, joint pain, abdominal pain, weight loss, or any new lumps. The review of systems was also noncontributory. The patient arrived in Qatar 18 months ago and did not travel since then. He worked as a farmer but did not have any contact with animals and he denied consumption of unpasteurized dairy products. On examination, the patient was febrile (T max 39.6C tympanic) and looked underweight, had a blood pressure of 97/49 mmHg, pulse rate of 82 beats per minute, and respiratory rate was 18 per minute. The meningeal signs were negative, and the only other positive finding was splenomegaly. Initial laboratory investigations showed pancytopenia, elevated liver enzymes, high C‐reactive protein (CRP), very high ferritin level, and a borderline low vitamin B12 level. In addition, laboratory screening for malaria, brucella, and autoantibodies were negative (Table 1). Ultrasonic examination of the abdomen and computed tomography of the chest and abdomen revealed hepatosplenomegaly (liver span 18 cm, spleen span 23 cm) and diffuse sclerosis of the axial skeleton. Chest X‐ray and echocardiogram were reported as normal, and the patient received cyanocobalamin replacement. On Day 9 postadmission, the patient was persistently febrile despite intravenous antibiotics and negative blood cultures, so we proceeded for bone marrow examination for aspirate, trephine biopsy, and culture examination. Three days later, the leishmania antibody was reported as positive by the ELISA method with an antibody titer of 1:64. Finally, bone marrow examination showed Leishman Donovan bodies both intracellularly and extracellularly in addition to active hemophagocytosis (Figure 1).

**TABLE 1:** Summary of clinical investigations during hospitalization.

Laboratory test	Value at admission	Value after 48 h of treatment	Normal range
Hb (g/dL)	8.2	8.0	13–17
RBC count (*10^6^/uL)	3.1	3.2	4.5–5.5
MCV (fL)	80.4	80	83–101
RDW (%)	13.6	14.9	11.6–14.5
WBC (*10^6^/uL)	1.6	2.1	4–10
ANC (*10^6^/uL)	0.8	0.8	2–7
Lymphocytes (*10^6^/uL)	0.7	1.2	1–3
Eosinophils (*10^6^/uL)	0	0	0–0.5
Basophils (*10^6^/uL)	0	0.01	0.02–0.1
Platelet (*10^6^/uL)	53	110	150–400
Urea (mmol/L)	4.3	6.5	2.5–7.8
Creatinine (umol/L)	88	95	62–106
Sodium (mmol/L)	131	136	133–146
Potassium (mmol/L)	3.4	4.7	3.5–5.3
Bilirubin (umol/L)	6	5	0–21
ALT (U/L)	69	159	0–41
AST (U/L)	129	134	0–40
CRP (mg/L)	83	42	0–5
PT (s)	13	‐	9.7–11.8
PTT (s)	44	‐	24.6–31.2
Fibrinogen (g/L)	3.45	‐	1.7–4.2 g/L
Retic count (*10^3^/uL)	42	‐	50–100
Retic (%)	1.5	‐	0.5–2.5
Ferritin (ug/L)	26,294	9776	38–270
Vitamin B12 (pmol/L)	127	‐	145–596
LDH (U/L)	870	618 U/L	135–225
Iron (umol/l)	4	‐	6–35
TIBC (umol/L)	39	‐	45–80
Iron saturation (%)	10	‐	15–45
ANA	Negative		
RF	Negative		
Anti‐CCP	Negative		
QuantiFERON TB test	Negative		
Brucella serology	Negative		
CMV PCR	Negative		
EBV PCR	Negative		
Parvovirus PCR	Negative		
Malaria smear	Negative twice		
Peripheral smear	Pancytopenia with normochromic anemia, neutropenia, lymphopenia, and moderate thrombocytopenia. No immature cells. Toxic features with a shift to the left		

At this stage, the diagnosis of visceral leishmaniasis with secondary HLH was made, and we decided to start anti‐leishmaniasis treatment with amphotericin B (3 mg/kg for 5 days, then one dose at 14 days and one dose at 21 days), and not to administer immunosuppressive therapy for HLH at the current time. During the consequent 48 h, the fever subsided completely, and his laboratory investigations showed improvement particularly with decreasing serum ferritin level (from 26,294 ug/L at baseline to 9776 ug/L after 2 days of amphotericin B), decreased CRP, and rapid increase in platelet count.

## 3 DISCUSSION

We described a case of fever of unknown origin (FUO) that turned out to be VL complicated by HLH. The nonspecific manifestations of VL and HLH alongside the rarity of VL in Qatar challenged our diagnostic approach. However, with the timely initiation of proper anti‐leishmania treatment, there was a dramatic response in clinical and laboratory parameters, and we managed to avoid the use of the more toxic treatment of HLH.

FUO is often referred to as a febrile illness with unclear etiology despite extensive evaluation. The most common underlying etiologies include infections, malignancy, and autoimmune systemic diseases.^7^ Nevertheless, the rate of infection as a cause of FUO is rapidly declining due to advancements in microbial detection.^8^ Although VL is known to present as a febrile illness, the delay in diagnosis could be attributed to the nonspecific initial laboratory investigations and the low index of suspicion for VL given the unexpectedly prolonged incubation period. Although this patient came from an endemic area of leishmaniasis, the incubation period of 18 months is longer than expected. According to Jeronimo et al., the incubation period for VL is often between 2 and 6 months, but that might vary between a few weeks to several years.^7^ In addition, it is believed that leishmania protozoa might persist lifelong in patients with asymptomatic infection, and later manifest during periods of immunosuppression.^9^ This finding highlights the importance of keeping VL as a differential diagnosis for fever with pancytopenia in patients coming from certain areas of the world even in the absence of recent travel history.

A definitive diagnosis of VL is established by identifying the organism in tissues or smears. The Infectious Disease Society of America (IDSA) and the American Society for Tropical Medicine and Hygiene (ASTMH) clinical practice guidelines issued in 2016 recommended bone marrow aspiration as the preferred site for diagnostic samples. They also advised that serological testing should only be used to diagnose suspected patients with VL who have negative or nonconclusive histopathological, microbiological, or molecular results. This recommendation could be justified by the fact that anti‐leishmania antibodies may remain detectable for years after treatment, and they also could be falsely negative in patients who are immunocompromised.^10^ Therefore, attending physicians should carefully interpret serological markers in patients with suspected VL.

As in this case, the clinical manifestations of VL and HLH largely overlap and not infrequently coincide in the same patient. Both conditions are associated with fever, pancytopenia, and hepatosplenomegaly.^11^ In a case series of 127 children with VL in Brazil, 27.5% of the patients had concomitant HLH.^6^ In comparison, another study in Germany found that only 2.1% of the children affected with VL fulfilled the criteria for HLH.^12^ This discrepancy in the prevalence of HLH in VL could be attributed to different levels of infection rate, severity, and diagnostic capabilities among various locations in the world. Notably, in a systematic review by Rajagopala et al. on 56 patients of HLH secondary to VL, around two third of the cases responded to leishmania‐specific treatment alone with only one‐third requiring adjunctive therapy mostly with delayed treatment.^13^ According to Rajagopala et al, VL‐related HLH is potentially underdiagnosed due to the overlapping nature of both conditions, in addition to the possibility of negative bone marrow examination at the beginning of the disease course due to low microbial load or patchy involvement.^13^ However, the dramatic response to anti‐leishmania medications alone was consistent with our observation in this case report.

We used liposomal amphotericin B to treat VL as recommended by IDSA/ASTMH.^10^ Amphotericin B disturbs leishmania plasma membrane by interacting with ergosterol precursors. Compared to the conventional form of amphotericin B, the lipid formula of amphotericin B is associated with a favorable side effects profile in terms of renal and other toxicities as well as enhanced penetration to tissues and macrophages infected with leishmania.^14^ Furthermore, animal studies showed persistent therapeutic levels of liposomal amphotericin B in the liver and spleen of infected mice with VL for at least 14 days after the loading doses.^15^

## 4 CONCLUSION

We presented a case of VL complicated by HLH which responded solely to anti‐leishmania treatment. A high index of suspicion should be maintained for VL in patients presenting with HLH features coming from endemic areas. Diagnostic tests for VL should include microbiological or molecular isolation of the organism with serological testing only complementing the workup. Liposomal amphotericin B is effective for VL with a favorable side effect profile.

## AUTHOR CONTRIBUTIONS

**Anwar I Joudeh:** Conceptualization; data curation; formal analysis; methodology; project administration; software; writing – original draft; writing – review and editing. **Hussein A Elsiddig Awadelkarim:** Data curation; formal analysis; investigation; writing – original draft. **Mohammadshah Isam Gul:** Data curation; investigation; writing – original draft; writing – review and editing. **Mahmoud Elayana:** Data curation; investigation; writing – original draft; writing – review and editing. **Dina Soliman:** Data curation; investigation; resources; writing – review and editing. **Aliaa Amer:** Data curation; investigation; resources; writing – original draft. **Musaed Alsamawi:** Conceptualization; data curation; formal analysis; investigation; methodology; project administration; resources; validation; visualization; writing – review and editing.

## FUNDING INFORMATION

The authors have not declared a specific grant for this research from any funding agency in the public, commercial, or not‐for‐profit sectors. Open Access fee was provided by Qatar National Library.

## CONFLICT OF INTEREST STATEMENT

The authors have no conflict of interest to declare.

## ETHICS STATEMENT

This work was conducted in accordance with the Declaration of Helsinki (1964).

## CONSENT

Informed written consent was obtained from the patient for publication of this case report and accompanying images without the patient identifying information.

## PERMISSION TO REPRODUCE MATERIAL FROM OTHER SOURCES

Not applicable.

## Data Availability

Data can be obtained from the corresponding author upon request.
